# Chromosome 12q13.13q13.13 microduplication and microdeletion: a case report and literature review

**DOI:** 10.1186/s13039-017-0326-4

**Published:** 2017-06-19

**Authors:** Jie Hu, Zhishuo Ou, Elena Infante, Sally J. Kochmar, Suneeta Madan-Khetarpal, Lori Hoffner, Shafagh Parsazad, Urvashi Surti

**Affiliations:** 1 Center for Clinical Genetics and Genomics, Pittsburgh Cytogenetics Laboratory, Magee-Womens Hospital of UPMC, Pittsburgh, PA 15213 USA; 2 Department of Obstetrics, Gynecology & Reproductive Sciences, University of Pittsburgh School of Medicine, Pittsburgh, PA 15213 USA; 3 Department of Genetics, Children’s Hospital of Pittsburgh of UPMC, Pittsburgh, PA 15224 USA; 4 Department of Pathology, University of Pittsburgh School of Medicine, Pittsburgh, PA 15213 USA

**Keywords:** 12q13.13 Microdeletion/Microduplication, Array *CGH*, *HOXC*, *SPT7*, *SP1*

## Abstract

**Background:**

Duplications or deletions in the 12q13.13 region are rare. Only scattered cases with duplications and/or deletions in this region have been reported in the literature or in online databases. Owing to the limited number of patients with genomic alteration within this region and lack of systematic analysis of these patients, the common clinical manifestation of these patients has remained elusive.

**Case presentation:**

Here we report an 802 kb duplication in the 12q13.13q13.13 region in a 14 year-old male who presented with dysmorphic features, developmental delay (DD), mild intellectual disability (ID) and mild deformity of digits. Comparing the phenotype of our patient with those of reported patients, we find that patients with the 12q13.13 duplication or the deletion share similar phenotypes, including dysmorphic facies, abnormal nails, intellectual disability, and deformity of digits or limbs. However, patients with the deletion appear to have more severe deformity of digits or limbs.

**Conclusions:**

Deletion and duplication of the 12q13.13 region may represent novel contiguous gene alteration syndromes. All seven reported 12q13.13 deletions and three of four duplications are de novo and vary in size. Therefore, these genomic alterations are not due to non-allelic homologous recombination.

## Background

There are only a handful of patients with a copy number variation (CNV) in the 12q13.13 or 12q13.13q13.2 region reported in the literature. Due to the variable sizes and number of genes involved in these CNVs, the common clinical features of these patients have not been identified. In the present study, we report one patient with a small 12q13.13q13.13 duplication and review genomic alterations and clinical features of additional four patients with a duplication and seven patients with a deletion, all of which are reported in the literature or in the DECIPHER (Database of genomic variation and phenotype in humans using ensembl resources) and dbVar (genomic structural variation) databases. The common clinical features are detailed. The duplication and deletion of the 12q13.13q13.2 region may represent novel microdeletion and duplication syndromes.

## Case presentation

**Fig. 1:**
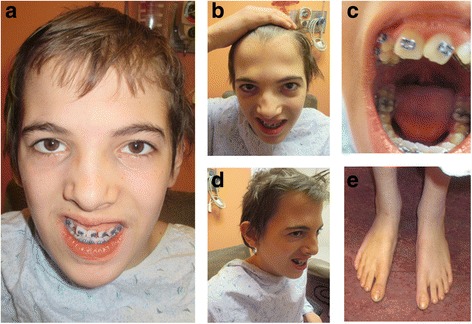
Patient at age 14 years and 8 months of age. **a** and **b** *:* facial features of the patient including long and narrow face, high arched heavy eyebrows with medial flare, hypertelorism, moles under left eye, broad nasal bridge and tip, short philtrum; **c** *:* bifid uvula and abnormal tooth; **d** *:* a side view of the patient showing the low-set ear; **e** *:* abnormal toenails

Our patient is a 14 year 8-month-old male with dysmorphic features and a history of developmental delay, learning difficulties and disruptive behaviors. He was born at 35 weeks gestation to a 21-year-old gravida 1, para 0 mother. The mother denied any use of medications. Fetal ultrasounds were normal. His birth weight was 2637 g. He had mild jaundice at birth and remained in the hospital for 5 days due to breathing difficulties. He sat at 1 year of age, walked between 13 or 14 months of age, and did not speak any words until 3 and a half years of age. He had some regression of learning according to the family. At the age of 8 years he was evaluated by the Child Development Unit and diagnosed with Attention Deficit/Hyperactivity Disorder (ADHD). Genetic evaluation was performed on this patient at the age of 14 years and 8 months. At the time of the genetic evaluation, his weight was 47.2 kg (20th %ile), height was 162 cm (22nd %ile), and head circumference was 53.2 cm (30th %ile). He had dolichocephaly with prominent occiput and a long and narrow face. His eyebrows were quite heavy with medial flare. His scalp hair was somewhat sparse and fine textured. He had telecanthus, with outer canthal distance (OCD) 9.6 cm (95th %ile) and inner canthal distance (ICD) 3.9 cm (>97th %ile). The cartilage of his ears was very soft and with Darwinian tubercle bilaterally. He had esotropia, narrow and short philtrum, bifid uvula, high palate, very broad nose in the middle as well as on the tip, and receding anterior hairline with widow’s peak. His genitalia was barely at the beginning of Tanner II. He also had hyperconvexed nails, collapse of arches of his feet, slight scoliosis, and quite large-looking elbows with prominent carrying angles. His skin was dry with eczema on the dorsum and numerous moles, especially under his left eye. At the time of evaluation, he was in the eighth grade but was in special education classes working at a second grade level. The representative photos of this patient are shown in Fig. 1.

The mother of the patient is 36 years old with learning disabilities. As a child, she had developmental delay and had speech therapy, occupational therapy and physical therapy. She finished high school and is attending school to become “a nurse”. The maternal grandmother also had developmental delays, special education, mental health concerns, and required speech therapy. The patient’s father is in his 40’s. He had delays and had learning disabilities. The patient’s 7-year-old and 5-year-old maternal half-sisters had no concerns reported.

## Methods

Microarray analysis was performed on purified DNA extracted from peripheral blood samples using Agilent’s SurePrint G3 CGH + SNP microarray (4x180K ISCA design) platform. The array analysis followed the standard manufacturer protocols. Parental analysis was not performed because of unavailability of the samples.

## Results

**Fig. 2:**
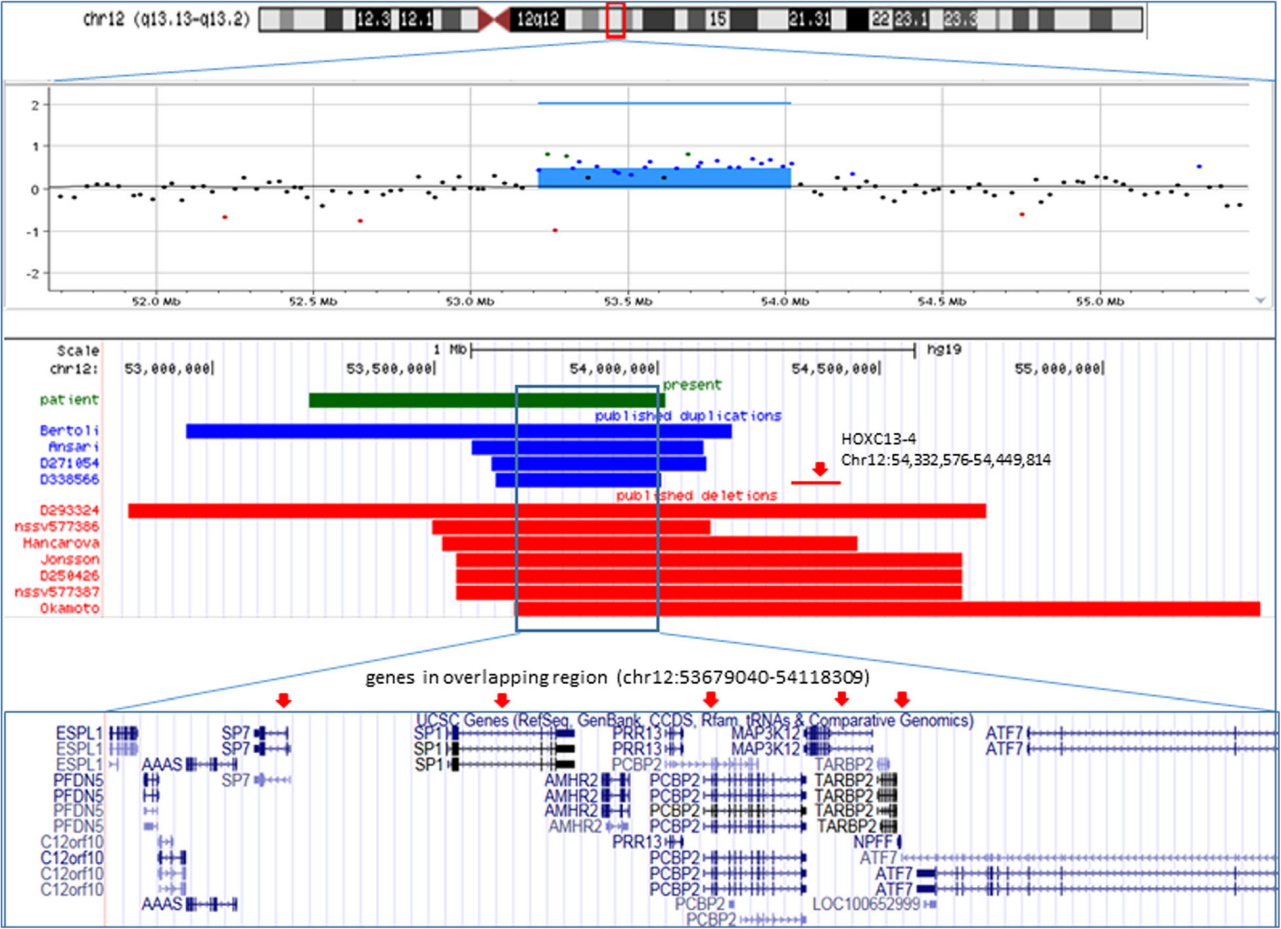
Chromosome 12q13.13 or 12q13.13q13.2 duplications and deletions found in our patient and previously reported patients. The *top panel* shows the ideogram of chromosome 12 with the 12q13.13q13.2 region marked in a *small red box*. The *scatter plot* of the Agilent array-CGH data shows an 802 kb microduplication of 12q13.13 in our patient. The schematic representation in the *middle panel* shows a comparison of the duplications and deletions involving the 12q13.13 or 12q13.13q13.2 region in the 11 reported patients and our patient. The *green bar* represents the duplication detected in our patient; the *blue bar bars* represent duplications identified in previously reported patients; the *red bars* represent deletions identified in previously reported patients. The common region shared by the deletions and the duplications is shown in a *box*. The UCSC genes in the overlapping region are shown in the *bottom panel*. The *red arrows* point to the candidate genes

The whole genome CGH + SNP microarray analysis detected a gain in copy number in the 12q13.13 region (chr12:53,304,719–54,018,772, hg19) of the long arm of chromosome 12, encompassing at least 802 kb and containing 21 OMIM genes (*KRT18, EIF4B, LOC283335, TENC1, SPRYD3, IGFBP6, SOAT2, CSAD, ZNF740, ITGB7, RARG, MFSD5, ESPL1, PFDN5, C12orf10, AAAS, SP7, SP1, AMHR2, PRR13, PCBP2, MAP3K12, TARBP, NPFF, ATF7*). The array findings are shown in the top panel of Fig. 2.

## Discussion

### Clinical features of patients with 12q13.13 duplication

Our patient had an 802 kb interstitial duplication in the 12q13.13q13.13 region. He presented with mild ID, ADHD, language difficulty and dysmorphic features including dolichocephaly, prominent occiput, bifid uvula, high arched palate, long and narrow face, high arched heavy eyebrows with medial flare, hypertelorism, esotropia, broad nose bridge and tip, short philtrum, and receding anterior hairline. He also had coned-shaped epiphyses of distal phalanges of the 2nd-5th digits, hyperconvex nails, stubby thumbs and collapse of arches of both feet.

Four patients with a similar duplication in this region were reported in the literature ([1, 2], DECIPHER 271054; DECIPHER 338566). The genome coordinates and size of the duplications, as well as phenotypes of all four reported patients and our patient are summarized in Table 1. The position of the duplications found in these patients are illustrated in the middle panel of Fig. 2. All five patients with duplication of the 12q13.3 region had ID, DD or language difficulties and dysmorphic facial features. The first patient, with a 1.2 Mb duplication in the 12q13.13q13.2 region, was reported by Bertoli et al. [2]. The duplication interval in this patient completely covers the region of duplication in our patient. Our patient and the first reported patient share some facial features including craniofacial anomalies, trigonocephaly or dolichocephaly, cleft palate or high arched plate and bifid uvula, narrow face and heavy eyebrows. In addition, mild hand and foot anomalies were also seen in our patient and the first reported patient. Some similarities with Wolf–Hirschhorn syndrome were described in Bertoli’s patient, such as high nasal bridge, shallow orbits, and hypertelorism, but these were not seen in our patient. It is noted that a patient with a larger duplication (5 Mb) of this region was also reported to have Wolf-Hirschhorn syndrome-like phenotype [3]. Therefore, the variable phenotypes between our patient and the first reported patient are likely due to the size of the duplication and the genes that are involved. The second previously reported patient with a 520 kb duplication in the 12q13.13 region, was reported to have a Cornelia de Lange (CdLS)-like phenotype [1]. The third and the fourth patients reported in the DECIPHER database (patients: 271,054 and 338,566) were described to have intellectual disability. In addition, the patient (271054) also had abnormal facies. No other details of the clinical features were reported in those three patients. However, it is well known that the typical facial features for CdLS patients include arched eyebrows and synophrys, some with upper limb anomalies, and small, widely spaced teeth, which are similar features observed in patients with deletion/duplication of chromosome 12q13.13 [4].

**Table 1:** Laboratory findings and clinical features of 5 patients with 12q13.13 duplication

Features	Present	Bertoli [2]	Ansari [1]	DECIPHER 271054	DECIPHER 338566
Age/Gender	14/M	6/F	NA/F	14/M	9/F
Size	802 Kb	1.2 Mb	520 Kb	484 Kb	370 Kb
Chromosome regions	12q13.13	12q13.13q13.2	12q13.13	12q13.13	12q13.13
Genomic coordinates					
(hg18)^a^		51,227,241–52,353,011			
(hg19)	53,217,136–54,018,772	52,940,974–54,166,744	53,582,733–54,102,733	53,627,092–54,111,152	53,637,649–54,007,964
# of genes	27	57	18	16	17
*HOXC* cluster	None	None	None	None	None
*SP1, SP7*, *NPFF, MAP3K12, PCBP*	Yes	Yes	Yes	Yes	Yes
Inheritance	Unknown	De novo	Unknown	De novo	De novo
Developmental Anomalies	ID, ADHD, language difficulty	ID, language difficulty	DD, ID	ID	ID
Craniofacial anomalies	Dolichocephaly, prominent occiput, bifid uvula, high arched palate.	Microcephaly, trigonocephaly cleft palate			
Dysmorphic facial features	Long and narrow face, high arched heavy eyebrows with medial flare, hypertelorism, esotropia, broad nasal bridge and tip, short philtrum, receding anterior hairlines, auricular tubercle	Long face, high arched eyebrows, prominent glabella, hypertelorism, prominent eye, lagoph-thalmos, shallow orbits, epicanthal folds, hypoplastic nasal alae, high nasal bridge, low-set ears, thin upper lip	CdLS-like phenotype	Abnormal face (no details described)	
Hand and foot anomalies	hyperconvex nails, stub thumbs, collapse of arches of feet	Small hands and feet, 2nd toe clinodactyly			
Skeletal anomalies	Mild scoliosis, coned-shaped epiphyses of distal phalanges of 2nd-5th digits				
Other	Dry skin, eczema on dorsum	Ataxia walk, corneal sclerosis, progeroid hands			

*ADHD* attention deficit hyperactivity disorder, *CdLS* Cornelia de Lange Syndrome, *DD* developmental delay, *ID* intellectual disability;

^a^hg18 nucleotide coordinates in published patients are converted into hg19 nucleotide coordinates

It is interesting to note that deletions of this region were also reported in seven patients ([5–7], DECIPHER patients: 293,324 and 250,426; dbVar: nssv577386 and nssv577387). A comparison of the location and size of the deletions and duplications are illustrated in the middle panel of Fig. 2. The reported laboratory findings and clinical features of those patients with deletion of the 12q13.13 region are summarized in Table 2. It appears that patients with either a deletion or a duplication of this region share some common phenotypic abnormalities, including intellectual disability, similar facial dysmorphism (long face, high palate/cleft palate) and mild limb anomalies (clinodactyly of toes and stub thumbs). However, compared to the patients with a duplication of this region, patients with a deletion of this region had more severe limb deformities including camptodactyly or flexion contracture of hand, which were reported in five of the seven patients. Club foot or valgus position of the feet was reported in one patient each (DECIPHER patient: 293,324, [6]). Moreover, heart defects which were seen in some of patients with deletion of the 12q13.13 region were not observed in patients with duplication of the same region. Other skeletal abnormalities, such as scoliosis and cone-shaped epiphyses of distal phalanges, reported in four of the seven patients with a 12q13.13 deletion ([5–7], DECIPHER patient: 250,426), were also seen in our patient who had a duplication in this region.

**Table 2:** Laboratory findings and clinical features of 7 patients with 12q13.13 deletion

Features	Okamoto 2011 [5]	Jonsson 2012 [6]	Hancarova 2013 [7]	DECHIPHER 293234	DECHIPHER 250426	dbVar: nssv577386	dbVar: nssv577387
Age/Gender	14/M	6/F	5/M	Unknown	Unknown	Unknown	Unknown
Size	1.7 Mb	1.13 Mb	0.9 Mb	1.93 Mb	1.13 Mb	624.86 Kb	988.42Kb
Chr. location	12q13.13q13.2	12q13.13	12q13.13	12q13.13	12q13.13	12q13.13	12q13.13
Genomic coordinates							
(hg18)^a^	51,965,307–53,642,659	51,834,791–52,971,391	51,801,299–52,737,892				
(hg19)	53,679,040–55,356,392	53,548,524–54,685,124	53,515,032–54,451,625	52,810,526–54,739,060	53,548,524–54,685,124	53,493,442–54,118,309	53,617,808–54,616,234
# of genes	61	33	21	81	42	34	44
*HOXC* cluster	9 of 9	9 of 9	9 of 9	9 of 9	9 of 9	0 of 9	9 of 9
*SP1, SP7*, *NPFF, MAP3K12* ***,*** *PCBP2*	Yes	Yes	Yes	Yes	Yes	Yes	Yes
Inheritance	De novo	De novo	De novo	De novo	De novo	De novo	De novo
Developmental anomalies	ID, language difficulty	Global DD, ADHD	Mild ID	ID	ID	DD	DD
Growth impairment	Short stature		Short stature, failure to thrive				
Dysmorphic facial features	Long face, broad nose, prominent ears, low-set ears, downslanting PF, strabismus, high palate	Bilateral epicanthal folds, depressed nasal bridge, slightly bulbous and anteverted nose, short philtrum	Microcephaly, long and narrow face, hypotelorism, enophthalmos, wide nasal root, long philtrum, low-set ears, fine hair	High palate, micrognathia, epicanthal folds	Anteverted nares, depressed nasal bridge, epicanthus, short philtrum	Other significant developmental or morphological phenotypes	Malar flattening
Hand and foot anomalies	PIP joint flexion, camptodactyly involving 3rd and 4th fingers, inflexible DIP joints of index fingers, adducted thumbs, dislocated radial heads	Flection contracture involving digits, hands, and elbows, ulnar deviation of both hands, valgus position of both ankles, short nails	Flection contracture involving 4th and 5th fingers (right hand), ulnar deviation of both hands, hyperlaxity of joints, hypoplastic abnormal nails	Distal flection contracture, small nail, talipes equinovarus	Short nail, ulnar deviation of the hand, flexion contracture		
Skeletal anomalies	Severe kyphosis, mild scoliosis	Short metacarpal and proximal phalangeal bones	Conical shaped distal phalanges, extremely long thorax, short lower limbs		Short phalanx of finger		Distal arthrogryposis
Other	Heart defect, bilateral inguinal hernias, hypodontia, persistent teeth	Umbilical hernia, pectus excavatum, subluxation at MCPIII,	Congenital heart defect, cryptorchidism, hyperelastic skin, abnormal palmar and plantar creases	Single transverse palmar crease	Umbilical hernia, pectus excavatum recurrent infections		

*ADHD* attention deficit hyperactivity disorder, *chr* chromosome, *DD* developmental delay, *DIP* distal interphalangeal, *ID* intellectual disability, *MCP* Metacarpophalangeal, *PF* palpebral fissure, *PIP* proximal interphalangeal

^a^hg18 nucleotide coordinates in previously reported patients are converted into hg19 nucleotide coordinates

### Candidate genes within the common deletion and duplication interval

There are at least 16 genes within the common overlapping region. A few of these genes are expressed in the central nervous system and/or likely to be dosage sensitive, or reported to be associated with disease by animal studies. These genes could be candidate genes for patients with deletion or duplication in this region.

The transcription factor gene (*SP1*) is most likely to be dosage sensitive (haploinsufficiency score: 0.81%) [DECIPHER]. The protein encoded by the *SP1* gene is a zinc finger transcription factor that binds to GC-rich motifs of many promoters and is then involved in a variety of cellular processes such as cell growth, apoptosis, differentiation and immune responses, DNA damage response, and chromatin remodeling (provided by RefSeq, Nov 2014). The *SP7* gene (haploinsufficiency score: 14.4%) encodes a bone specific transcription factor (osterix) which regulates osteogenesis and bone formation during embryonic development [8]. Niger et al. [9] reported that the activity of osterix (Osx/Sp7) is influenced by *Sp1*. The *sp7* ^−/−^ mutation in zebra fish causes a generalized delay in osteoblast maturation, while heterozygous zebra fishes (*sp7* ^+/^−) are not appreciably different from wild type siblings, however; they exhibit an increase in the variance of craniofacial shape [10]. Dolichocephaly and trigonocephaly are seen in our patient and the first reported patient with a duplication in the 12q13.13 region. Moreover, ‘long’ or ‘long and narrow’ facies are a common feature in patients with a deletion or duplication in the 12q13.13 region. It is known that a homozygous single base pair deletion (c.1052delA) in the *SP7* gene resulted in a child with osteogenesis imperfecta born to heterozygous carrier parents who were phenotypically normal [11]. However, the clinical features of the patients with heterozygous contiguous gene deletion/duplication involving both the *SP1* and *SP7* genes have not been investigated. Our study indicates that patients with either a deletion or a duplication of the 12q13.13 region, involving the *SP1* and *SP7* genes, share some common clinical features with those patients who have the homozygous *SP7* mutation, such as short stature, high-arched palate, mild bone deformities, mild scoliosis, and delayed motor milestones. Whether concurrent dosage changes of the *SP1* and *SP7* genes cause some of the abnormal features in these patients is unclear at present.

The *HOXC* genes are not within the common duplication interval. However, six of seven reported patients with the 12q13.13 deletion involve the *HOXC* gene cluster, which are thought to be the candidate genes for the development of skeletal anomalies and limb deformities [5–7]. Mutations or deletions involving other *HOX* genes have been well documented in developmental disorders in both humans and mice, particularly limb anomalies [12]. Our earlier study found that patients with deletion of the 3′ portion (distal) of the *HOXC* cluster including *HOXC11, HOXC10, HOXC9, HOXC8, HOXC6, HOXC5* and *HOXC4* showed no skeletal anomalies or obvious digit anomalies [13]. A recent study of multiple members from multiple generations in four families showed that the small deletions in the upstream noncoding region, with or without deletion of *HOXC13 and HOXC12,* segregated with limb deformities [14], which indicates that a putative regulatory element within that region might be deleted in those patients. Variable expressivity including club foot, vertical talus, hammertoes and syndactyly was reported in those patients. Therefore, it is possible that a deletion of the 5’of *HOXC* gene cluster may contribute to a relatively more severe phenotype in patients with the 12q13.13 deletion.

Developmental delay and intellectual disability were found in all patients with a 12q13.13 or 12q13.13q13.2 deletion/duplication. The common region for the copy number alterations in this region contains a few genes expressed in the central nervous system (*NPFF, SP1, and MAP3K12*). In addition to the *Sp1* gene the heterogeneous nuclear riboprotein E2 (*PCBP2*) gene is also predicted to be dosage sensitive with a haploinsufficiency score of 4.18% [DECHIPHER]. Although the precise phenotype-genotype correlation for these genes cannot be established, the dosage changes of these genes may contribute to the neurodevelopmental anomalies in these patients. Although parental studies were unavailable for our patient, 10 of the 11 reported deletions or duplications were found to be de novo which supports the likely pathogenic nature of these CNVs.

## Conclusions

We report one more patient with a chromosome 12q13.13 q13.13 duplication. The phenotype of this patient shows great similarity with the previously reported patients who have either a duplication or a deletion in this region, including a long face, high nasal bridge, high arched eyebrows, cleft palate, skeletal anomalies, limb deformity, language difficulty and intellectual disability. However, patients with deletion of this region have more severe skeletal anomalies, limb deformities and heart defects. Additional cases are needed to confirm these syndromes.

## Abbreviations

*aCGH*: Array comparative genomic hybridization
ADHD: Attention deficit hyperactivity disorder
CdLS: Cornelia de Lange Syndrome
Chr: Chromosome
CNV: Copy number variation
*DD*: Developmental delay
DECIPHER: Database of genomic variation and phenotype in humans using ensembl resources
DIP: Distal interphalangeal
ICD: Inner canthal distance
ID: Intellectual disability
Kb: Kilobases
Mb: Megabases
MCP: Metacarpophalangeal
OCD: Outer canthal distance
OMIM: Online Mendelian Inheritance in Man
PF: Palpebral fissure
PIP: Proximal interphalangeal
